# Improving Nasal Airflow with a Novel Nasal Breathing Stent

**DOI:** 10.3390/dj10050081

**Published:** 2022-05-11

**Authors:** Hiroshi Suzuki, Arisa Sawa, Tatsuo Yagi, Yoshihiro Iwata, Toshiyuki Nakayama, Chin-Moi Chow, Osamu Komiyama

**Affiliations:** 1 Department of Oral Function and Fixed Prosthodontics, Nihon University School of Dentistry at Matsudo, Chiba 271-8587, Japan; sawa.arisa@nihon-u.ac.jp (A.S.); iwata.yoshihiro@nihon-u.ac.jp (Y.I.); toshix41@gmail.com (T.N.); osamu.komiyama@nihon-u.ac.jp (O.K.); 2 Department of Physical Reaction, School of Physical Education, Tokai University, Tokyo 259-1292, Japan; yagitatsuo@gmail.com; 3 Sleep Research Group, Charles Perkins Centre, University of Sydney, Sydney, NSW 2006, Australia; chin-moi.chow@sydney.edu.au; 4 Sydney School of Health Sciences, Faculty of Medicine and Health, University of Sydney, Sydney, NSW 2050, Australia

**Keywords:** obstructive sleep apnea, nose obstruction, nasal dilator, airflow, peak nasal inspiratory flow rate

## Abstract

Nasal obstruction requires close attention, as it is a risk factor for obstructive sleep apnea (OSA). This study evaluated airflow rates of our newly designed nasal breathing stent (NBS) compared with those of existing nasal dilators in 10 adult men. We hypothesized that the NBS would expand the nasal passage more than the other nasal dilators by means of airflow measurements. We compared airflow measurements between the NBS and three existing appliances and no appliance. Velocity measurements were recorded by analyzing 499 videographic images when each appliance was placed next to a steam generator at 0, 5, and 10 mm from the outlet port for airflow visualization. The peak nasal inspiratory flow (PNIF) rate was measured using an inspiratory flow meter. The NBS resulted in significantly higher airflow velocity measurements at all distances from the outlet port and a higher PNIF rate than the other appliances. Thus, the NBS offers a significantly decreased resistance to air movement compared with other appliances. Future in-depth investigations are required to demonstrate the use of NBS as a nasal dilator in conjunction with continuous positive airway pressure/oral appliance treatments in patients with OSA.

## 1. Introduction

Nasal obstruction is a symptom of an underlying disorder and can be caused by partial or complete blockage of the air passage. Structural abnormality of the nasal septum, chronic sinusitis and allergic rhinitis are some common causes. Among the various risk factors responsible for obstructive sleep apnea (OSA), nasal obstruction (NO) demands close attention. NO not only induces oral breathing that exacerbates apnea but is also directly linked to low adherence to continuous positive airway pressure (CPAP) treatment or a decrease in the frequency of oral appliance (OA) use [1,2,3,4]. Hence, the treatment of NO is key to the treatment of OSA.

NO treatment methods vary according to the primary underlying disease. Drug therapy is a common approach for variable NO caused by allergic diseases [5], whereas surgical treatment is often chosen to fix NO caused by nasal septal deviation and in patients with drug resistance [6]. Hellings and Trenite (2014) reported that enlarging the nasal valve with a nasal dilator helped improve NO [7].

A nasal dilator is inserted into the nose or applied externally to improve airflow dynamics and alleviate obstruction within the nasal valve. Its advantage lies in the simplicity of its use and relatively non-invasive nature. Although snoring can be reduced to a certain degree, one study reported skeptical results regarding its effects in improving sleep breathing parameters, such as the apnea-hypopnea index (AHI), sleep architecture, and daytime sleepiness assessed by the Epworth Sleepiness Scale [8]. Djupesland et al. reported subjective improvement in sleep and snoring but not in objective measures of sleep using polysomnography and snoring with the Breathe Right^®^ (BR) nasal dilator in a group of heavy snorers with mild OSA [9]. In a limited subgroup, the authors observed that participants with a nasal airway dimension of <0.6 cm^2^ (as measured by the total minimum cross-sectional area) and an AHI <10 showed a significant reduction in AHI, whereas participants with a nasal airway dimension of >0.6 cm^2^ and an AHI > 10 showed a significant increase in AHI [9]. Another study reported that the use of Nozovent^®^ nasal clips lowered the AHI, the severity of oxygen desaturation, and snoring noise in patients with habitual snoring and/or OSA. However, no subjective effects on arousal frequency or daytime sleepiness by using a visual analogue scale have been reported [10]. The ineffectiveness of these dilators in treating sleep-disordered breathing is not surprising given the limited amount of nasal expansion to alleviate obstructive apnea. However, a dilator that functions well and improves nasal airflow may play a pivotal role in keeping the airway patent during CPAP/OA treatment, as nasal breathing with a closed mouth is essential for the success of CPAP and OA treatments. This has led to the development of a novel nasal stent, the nasal breathing stent (NBS) which is functional and comfortable. In this study, as a proof of concept, we evaluated the aerodynamic effects of the NBS by comparing its velocity rates with those of three other nasal dilators, as each appliance offers varying levels of resistance to air movement. We hypothesized that the novel nasal stent would show accelerated airflow velocity and higher inspiratory nasal airflow via the peak nasal inspiratory flow (PNIF) compared with the existing appliances and a control condition (no appliance).

## 2. Materials and Methods

### 2.1. Participants

Ten adult men (mean age: 25.6 ± 0.97 years) without evident abnormalities of the oral cavity or maxillofacial structures were included in this study to measure the PNIF. Participants undergoing treatment for ear, nose, and throat diseases were excluded.

Prior to enrolment, written informed consent was sought from the participants. This study was approved by the Ethics Committee of Nihon University School of Dentistry at Matsudo (EC 18-015).

### 2.2. Nasal Dilators

**Figure 1:**
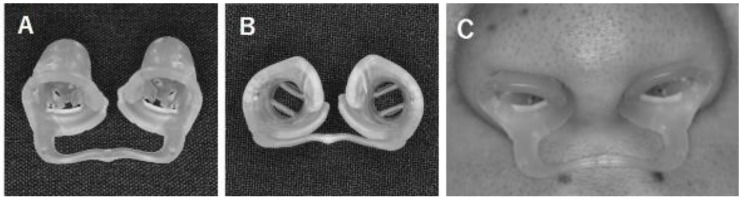
A novel nasal dilator; the nasal breathing stent (NBS). (**A**) Front view; (**B**) upper view; (**C**) NBS set.

We developed a new nasal dilator, the NBS. The designs that underly the NBS are as follows: (1) we used a polyester elastomer as the base material. This material is adequately flexible and resilient. It is also washable and does not irritate the nasal mucosa; (2) since the stent is rounded and smooth, it prevents damage to the nasal mucosa; (3) we implemented a lattice-type structure with an uneven pattern that offers efficient vaporization of nasal secretions/substances thus preventing them from entering the nasal mucosa during inspiration; (4) when worn, the NBS expands the nasal valve by pressing the depressor septi at the joint; (5) the NBS design facilitates airflow at the exits via enhanced diameter differences between the air entry and exit sections as well as the dual-plate structure of the exhaust plate at the outlet port. The dual-plate structure acts to rectify airflow issues thereby increasing velocity (Figure 1).

**Figure 2:**
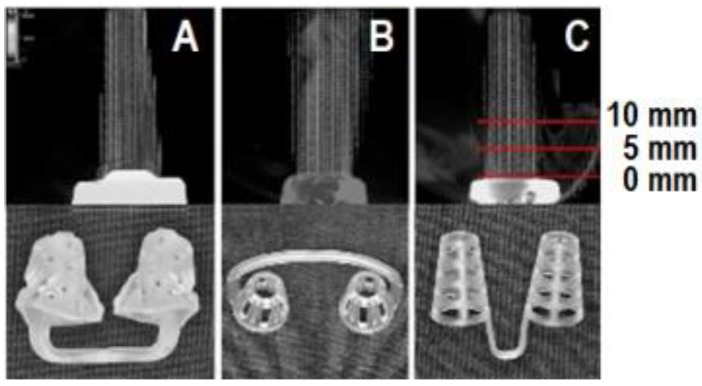
Images of airflow visualization. (**A**) The nasal breathing stent (NBS); (**B**) nose cones; (**C**) mute with hole. Measurements were taken at the appliance exit (0 mm), and at vertical distances of 5 mm and 10 mm from the exit.

In this study, the comparator dilators used were Max-Air Nose Cones^®^ (NC) (Sanostec Corp., Beverly Farms, MA, USA) and Mute with hole^®^ (MT) (Sleep Dreamz Co., Ltd., Ipswich, UK) (both dilators were meant for internal use) (Figure 2), and BR (an external nasal dilator, CNS Inc., Bloomington, MN, USA). The participants selected the nasal dilator size that was appropriate for them (usually medium or large).

### 2.3. Velocity Measurement by Visualization

We measured the airflow velocity with the NBS, NC, and MT at three sites, that is, at 0, 5, and 10 mm from the outlet port of the nasal dilator (Figure 2), to confirm the aerodynamic nature of the flow effects of each appliance. This test was used as the characteristics of airflow can be visualized. As steam passed through the appliance, air movement images were captured for airflow visualization (Supersonic Humidifier EL-C301, Senju Co., Ltd., Tokyo, Japan). The light source (PIV Laser G450, KATOKOKEN Co., Ltd., Isehara, Japan) emitted green light when steam passed through the appliance at an average speed of 2687.3 mm/sec. The airflow movement was captured using a high-speed camera (USB 3.0 High-speed Camera K5, KATOKOKEN Co., Ltd., Japan) at a sampling frequency of 8000 Hz for a motion picture of 0.000125 s/500 cuts to obtain the mean velocity at each measurement point. The airflow movement was also filmed without an appliance (‘no appliance’). BR was not evaluated since it was a nasal strip. A total of 499 videographic images were recorded for the airflow measurement analysis, as this number of shots represented the maximum taken to ensure a high degree of accuracy. FlowExpert2D2C software (KATOKOKEN Co., Ltd., Toyohashi, Japan; Figure 2) was used for automatic velocity analysis.

### 2.4. PNIF Measurement by a Flow Meter

**Figure 3:**
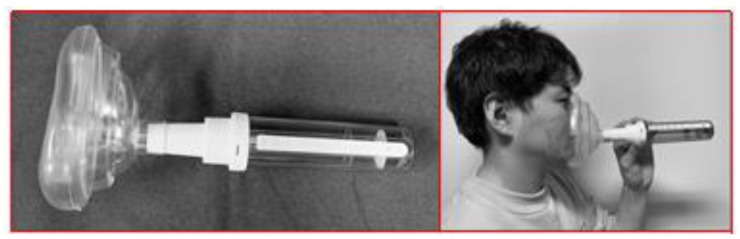
Photographs of the peak nasal inspiratory flow (PNIF) meter and its use.

PNIF measures the maximum inspiration amount per minute (L/min) during nasal breathing. It is a simple test and an easy way to measure airflow. We measured PNIF in awake participants with the NBS, NC, and MT by inserting them into the nasal cavity but applying the BR to the nasal bridge. This process permitted objective evaluation of inspiratory flow rates. For the control condition, PNIF was measured with ‘no appliance’. We recorded PNIF using an inspiratory flow meter (In-check™, Clement Clarke International Limited, Harlow, UK) (Figure 3). During the test, participants were instructed to inhale maximally through the nasal passage after exhaling completely in a sitting position with the mouth closed. The mean of three trials was recorded. The appliances were applied in a random order. As a safety check, an otolaryngologist examined the participants at the end of each session to assess the presence or absence of pain, discomfort, bleeding, or lacerations in the nasal cavity.

### 2.5. Statistical Analysis

One-way analysis of variance with repeated measures was used to compare airflow measurements obtained at three locations (0, 5, and 10 mm) for each appliance and PNIF evaluation. The Bonferroni correction was used for multiple comparisons. The significance level for each analysis was set at *p* < 0.05.

## 3. Results

### 3.1. Velocity Measurement by Visualization

The mean velocity was significantly higher with the NBS at each measurement site (0, 5, and 10 mm) than with ‘no appliance’, the NC, and the MT (*p* < 0.001). Moreover, the MT resulted in a significantly lower velocity than in the condition with ‘no appliance’ (Table 1).

### 3.2. PNIF Measurement by a Flow Meter

The mean PNIF value was significantly higher for the NBS than for ‘no appliance,’ the NC, MT, and BR (*p* < 0.05) (Table 2). Although four participants complained of discomfort with the NC and MT (related to inappropriate appliance size) during the post-session examination, none of the participants experienced any complications such as bleeding or laceration.

## 4. Discussion

We compared the flow dynamics of the NBS to those of ‘no appliance’, the NC, MT, and BR. This study evaluated the performance of the NBS through measurements of airflow dynamics using the flow visualization method, which enables velocity quantification in addition to PNIF measurement. We confirmed our hypothesis that NBS significantly accelerated airflow velocity and PNIF when compared with conventional nasal dilators and thus improved airflow through the nasal passage, as reflected in the PNIF.

In evaluating the aerodynamic effects of the NBS, we showed that the velocity was significantly higher with the NBS measured at 0, 5, and 10 mm from the outlet port of the nasal dilator than with other appliances. As steam was passed at a constant rate, the observed higher velocity suggests that the NBS offered lower resistance to air movement. Several studies have reported improvements in CPAP treatment adherence among patients with OSA using nasal strips [11,12]. However, this aspect has not been evaluated from the perspective of airflow velocity. The favorable outcomes of the flow visualization experiments on the NBS may be attributed to its design, which aims to provide aerodynamic efficiency via differences in the diameter at the air entry and exit sections and via the dual-plate structure of the exhaust plate at the outlet port. We were surprised that the MT showed a lower velocity than that observed with ‘no appliance’, suggesting that the MT offered sizable nasal resistance to air movement. It should be noted that the MT showed some improvement in PNIF compared with ‘no appliance’, but not in the in vitro model of velocity measurement by visualization. This difference may be explained by voluntary inhalation and the effort-dependent nature of the PNIF test. Thus, at high inhalation flow rates, the PNIF values increased for all of the nasal dilators used. In the visualization measurement, the speed at which the steam passed was held constant for all measurements. We included the MT in this study since it is one of the most commonly available items on the market, although no significant report in the literature could confirm this fact. For example, Snorepin^TM^ is a popular product on Amazon.com, with approximately 3000 reviews at the time of publishing this article (https://www.amazon.com/SnorepinTM-Solution-Conditions-Naturally-Effectively/dp/B000BABW5Q. Accessed 4 June 2018). A possible explanation for the lower velocity and lower PNIF with the MT is that the structural characteristics of a relatively large mesh may have allowed air leakage.

The analytical approach used for quantitative simulations is a widely used flow visualization method for fluid (liquid and gas) dynamics [13]. However, various external factors can affect the actual airflow conditions, which could lead to inconsistencies between the simulation results and analyzed data. Therefore, in our flow visualization experiment, a velocity measurement device was used to measure the airflow in a contactless manner by allowing visualization of the actual flow aerodynamics. The results indicated that this method of measurement enabled the evaluation of stent-type nasal dilators, offering a promising method for similar future investigations.

In the second series of experiments, in which the participants wore each appliance for the PNIF measurement, we investigated the effects of the appliances on inspiratory flow rates. The results indicated a significantly higher mean PNIF with the NBS than with the other appliances. PNIF is a measurement of the maximum inspiration amount per minute (L/min) during nasal breathing and is often used for objective evaluation of asthma or NO. The requisite equipment is inexpensive, small, and lightweight, allowing for easy measurement, which makes it practical for tasks [14,15]. Therefore, the significantly higher PNIF value for the NBS could provide evidence of its favorable effect, not only due to the ability of the nasal valve to expand but also due to the effective structural design that reinforces expansion caused by pressing the depressor septi at the joint. However, a limitation of PNIF measurements is that it is effort-dependent and does not correspond to spontaneous breathing. Rhinomanometry is a suitable method for measuring nasal airflow during normal inspiration and expiration through the nose. However, rhinomanometry cannot be performed using nasal stents.

The difference in the diameters of the air entry and exit sections of the NBS, as well as the dual-plate structure of the exhaust plate at the outlet port, averts a decrease in velocity at the exits. None of the participants had any issues linked to discomfort or pain during the post-session examinations of the nasal cavity, which may be attributed to the use of polyester elastomer (which is adequately flexible, resilient, and washable) as a comfortable base material, while the rounded design prevented damage to the nasal mucosa. The above-mentioned characteristics of the NBS may be the reason for this favorable result (a higher PNIF with the NBS than with the NC). Another reason may be that the NBS was designed to fit the noses of Asian individuals.

A previous study showed that the NC exhibited a higher PNIF than BR [16], which is similar to the results of our study. Some participants complained of discomfort with the NC or MT during the post-session examination interview. The issue of size is particularly significant with respect to eliciting a stable effect with stent-type nasal dilators. Further investigation regarding the available sizes of the NBS is essential for its practical use by patients of all races, ages, and sexes.

This study has some limitations. We investigated only two measures, namely airflow velocity (one of the performance features of the NBS) and the PNIF, the latter of which requires effort and was performed during the waking period. It is necessary to confirm whether increased, and accelerated inspiration can resolve pharyngeal obstruction. The next steps include documentation of objective measures of increased airway dimensions, for example, acoustic rhinometry, cephalometric radiographs, or ultrasound, followed by a clinical trial to evaluate the efficacy of the NBS in reducing nasal obstruction by examining the outcome measures of the AHI score or oxygen saturation during sleep. Further investigations are necessary to examine the effects of NBS alone or with CPAP/OA treatment during sleep.

In summary, our findings indicate that the NBS shows a significantly higher airflow velocity through visualization compared with the other appliances. The NBS also showed a significantly higher PNIF in the awake condition than that of the other appliances. Considering these results, we conclude that the NBS is a safe and effective nasal dilator to use in patients with OSA.

## Figures and Tables

**Table 1:** The change in velocity for each appliance.

Distance	No Appliance		NBS		Nose Cones		Mute with Hole		F	* p *	Multiple Comparison
	Mean	SD	Mean	SD	Mean	SD	Mean	SD			
0 mm	2687.3	41.1	3203.7	122.9	2748.9	140.2	2046.6	121.1	7534.4	<0.001	Mute with hole < No appliance < Nose Cones< NBS
5 mm	2686.4	36.2	3146.5	111.1	2967.8	82.5	2186.4	61.9	13,781.3	<0.001	Mute with hole < No appliance < Nose Cones < NBS
10 mm	2661.6	46.8	3180.1	82.5	2891.9	109.8	2324.4	62.5	12,144.3	<0.001	Mute with hole < No appliance < Nose Cones < NBS

All: *n* = 499; velocity unit: mm/h. Values are presented as the mean ± SD. NBS, nasal breathing stent; SD, standard deviation.

**Table 2:** Peak nasal inspirator flow of each appliance.

	No appliance		NBS		Nose Cones		Mute with hole		Breathe Right^®^			
	M	SD	M	SD	M	SD	M	SD	M	SD	F	* p *
PNIF	115	20.08	184.67	37.06	157	29.83	145.33	28.07	132.5	23	16.25	0.002

All: *n* = 10, PNIF, peak nasal inspiratory flow; unit: L/min. Values are presented as the mean ± SD. M, mean; SD, standard deviation; NBS, nasal breathing stent.

## Data Availability

Not applicable.
